# Roles and Molecular Mechanisms of Serum Exosomal miRNA-223 and miRNA-132 in Juvenile Idiopathic Arthritis

**DOI:** 10.7759/cureus.85809

**Published:** 2025-06-11

**Authors:** Xiaoying Chen, Wenting Li, Liping Li, Lei Ying, Xiaohui Liu, Jiangwei Ke

**Affiliations:** 1 Department of Clinical Laboratory, Children's Hospital of Jiangxi, Nanchang, CHN; 2 Department of Rheumatology and Immunology, Children's Hospital of Jiangxi, Nanchang, CHN

**Keywords:** exosomes, juvenile idiopathic arthritis, mirna-132, mirna-223, socs3, stat3

## Abstract

Introduction: The pathogenesis of juvenile idiopathic arthritis (JIA) has not yet been clarified and is closely related to persistent overactivation of the JAK/STAT signaling pathway. MicroRNA (miRNA)-223 (miR-223) and miRNA-132 (miR-132) might be involved in the development of JIA. However, the mechanism underlying the pathogenesis of JIA is unclear. In this study, we investigated the roles and molecular mechanisms of serum exosomal miR-223 and miR-132 in JIA.

Methods: Patients with systemic JIA were selected as the systemic group (sJIA), patients with polyarticular JIA and oligoarticular JIA were selected as the articular group (aJIA), and healthy children who underwent physical examinations at the same time were selected as the normal control group (NC). Exosomes were extracted from the serum, and the purified exosomes were subjected to electron microscopy to observe their particle morphology. The particle size distribution and concentration of the exosomes were detected by an N30E particle size analyzer. The expression levels of miR-223 and miR-132 in exosomes were quantitatively detected by the SYBR green method. The protein levels of STAT3 and SOCS3 were detected by Western blot.

Results: The expression level of miR-223 in serum exosomes of the sJIA group was significantly higher than in the aJIA group and the NC group (4.04±0.34 vs. 1.52±0.30, 0.88±0.17), and the difference was statistically significant (P<0.001). However, the expression level of miR-132 in serum exosomes of the sJIA group was significantly lower than in the aJIA group and the NC group (0.09±0.01 vs. 0.17±0.02, 0.94±0.08), and the difference was statistically significant (P<0.001). The expression levels of serum interleukin (IL)-6, IL-8, and IL-10 in the sJIA group were significantly higher than those in the aJIA group and the NC group; the difference was statistically significant (P<0.001). The IL-17 expression in the sJIA group and aJIA group was significantly greater than the expression levels in the NC groups (P<0.001). The expression level of miR-223 in exosomes was positively correlated with the expression levels of the clinical inflammatory factors IL-6, IL-8, IL-10, and IL-17 (P<0.001). However, the expression level of miR-132 in exosomes was negatively correlated with the expression levels of clinical inflammatory factors of IL-6, IL-8, IL-10, and IL-17 (P<0.001). The expression level of SOCS3 protein in both the sJIA and aJIA groups was significantly higher than in the NC group (0.25±0.05 and 0.21±0.03 vs. 0.10±0.02, respectively), and the difference was statistically significant (P<0.05). Regrettably, there was no significant difference yet in the expression level of STAT3 in the three groups (P>0.05).

Conclusion: miR-223 and miR-132 may be two potential new markers of JIA, providing new ideas for diagnostic tests and therapeutic interventions. But larger studies are needed to confirm these findings and assess their generalizability across diverse populations. miR-223 may promote inflammation in JIA patients by enhancing the JAK/STAT signaling pathway, while miR-132 may reduce inflammation in JIA patients by inhibiting the JAK/STAT signaling pathway. Its inhibitory effect may be closely related to SOCS3 through the JAK/STAT signaling pathway.

## Introduction

Juvenile idiopathic arthritis (JIA) is a common chronic connective tissue disease in childhood characterized by chronic joint synovitis that can be accompanied by multiple-organ functional impairments throughout the body and is one of the primary causes of disability and blindness in children [1]. The prevalence of JIA ranges from 15 to 400 cases per 100,000 people worldwide [2]. In recent years, the age of onset of JIA has gradually decreased, and the incidence of JIA has also increased [3]. In 2001, the International Federation of Rheumatic Diseases classified JIA into seven types, with systemic, polyarticular, and oligoarticular forms being the most common [1,2]. The onset of JIA is often unknown. Unfortunately, there are some difficulties in the early diagnosis of JIA and challenges in managing specific JIA subtypes due to the lack of specific biomarkers, which currently mainly depend on clinical and imaging findings. Moreover, the pathogenesis of JIA is not fully understood. Recent studies have indicated that JIA is an autoinflammatory disease. Due to persistent overactivation of the JAK/STAT signaling pathway, a large number of proinflammatory factors, such as interleukin (IL)-1, IL-6, IL-8, and IL-10, are produced at such high levels that patients with JIA are in a state of extreme inflammation, an important factor in JIA pathogenesis [4,5]. Tobias Schmidt et al. reported that in patients with JIA, once IL-6 binds to a receptor such as IL-6R or gp130 complex on the target cell membrane, its receptor dimerizes, resulting in autophosphorylation of JAK and activation of STAT3 into a dimer. Then STAT3 is translocated into the nucleus and directly promotes IL-6 transcription, forming a positive feedback loop. Finally, the JAK/STAT signaling pathway is continuously activated, resulting in the overexpression of IL-6 [5].

MicroRNAs (miRNAs) are a class of noncoding, single-stranded RNAs that are widely found in living organisms. By specifically binding to the 3' untranslated region (3' UTR) of a target mRNA, miRNAs can degrade the target mRNA or inhibit its translation process and participate in many basic biological processes, such as cell development, cell proliferation, differentiation, and the cell cycle [6]. Xiaolin Ma et al. have shown that many miRNAs, such as miRNA-16, miRNA-146a, miRNA-223, and miRNA-132, are expressed at different levels in the serum of JIA patients and are involved in the occurrence of JIA-related inflammation [7]. It has also been reported [8] that in JIA patients, multiple miRNAs, such as miRNA-21 and miRNA-19a, affect the regulation of the JAK/STAT signaling pathway. Xiaoxun Du et al. [9] have shown that miRNA-223 promotes the inflammatory response and induces cell injury of nucleus pulposus cells by acting on the JAK2/STAT1 pathway. As reported by Ya-Jun Song et al. [10], the downregulation of miRNA-132 expression can reduce the inflammatory response by inhibiting the JAK/STAT signaling pathway [10]. Nevertheless, the target genes of these miRNAs and the mechanism regulating the JAK/STAT signaling pathway remain unclear. To our knowledge, there was no report on how miRNA-223 and miRNA-132 affect the inflammatory response through the JAK/STAT signaling pathway in JIA.

Exosomes are the smallest type of extracellular vesicle and have a bilayer lipid membrane structure with diameters of approximately 30-100 nm. Exosomes carry large amounts of biological information, including proteins, lipids, and nucleic acids (e.g., mRNA, microRNA, and lncRNA), and are widely involved in intercellular communication, playing an important role in physiological and pathological processes. Exosomes can be isolated from a variety of body fluids, including urine [11], blood, saliva, breast milk, amniotic fluid, ascites, cerebrospinal fluid, bile, and semen [12]. Exosomes isolated from serum have important roles as potential biomarkers for pancreatic cancer [13], glioblastoma multiforme [14], gastric cancer [15], Parkinson's disease [16], and other diseases. Recent studies have focused on the roles of serum miRNAs in JIA. However, due to the complex composition of serum, the source of the miRNA may be unknown, which may have a certain impact on the detection results. Data have shown that miRNAs are stable in serum exosomes and that their composition is less complex than that in serum, meaning that serum exosomal miRNAs could be reliable disease biomarkers [17]. There are a few reports on the role of exosomes in JIA. Nirit Mor-Vaknin et al. [18] reported that DEK is secreted by synovial macrophages in a free form and via exosomes, and DEK can contribute directly to joint inflammation in JIA by generating immune complexes through high-affinity interaction between DEK and DEK autoantibodies. This study aims to test the hypothesis that serum exosomal miRNA-223 and miRNA-132 are differentially expressed in systemic JIA (sJIA) and the articular JIA (aJIA) (polyarticular and oligoarticular JIA) patients compared to healthy controls and that these expression levels correlate with disease activity and the expression of JAK/STAT pathway components. We aimed to explore the possible molecular mechanisms by which miRNA-223 and miRNA (miR)-132 affect the JAK/STAT signaling pathway in JIA and to provide new biomarkers for the diagnosis of JIA, as well as new ideas or new directions for the pathogenesis and biological targeted therapy of JIA.

This article was previously presented as a meeting abstract at the 29th Pediatric Academic Congress of the Chinese Medical Association on October 10, 2024.

## Materials and methods

Procedures

Selection of Research Objects

In the Rheumatology and Immunology Department of Jiangxi Children's Hospital, 60 patients with sJIA and 23 patients with aJIA were selected from September 2022 to December 2023. 

All cases that met the 2001 classification criteria for JIA of the International League of Associations for Rheumatology (ILAR) and were explicitly diagnosed as new patients with sJIA or aJIA were included in the study. Patients with JIA already been treated and patients with other joint diseases were excluded.

In addition, 20 healthy individuals (healthy control group, NC group) were selected from the child health care department of our hospital during the same period. This study was approved by the Hospital Medical Ethics Committee (Approval No. JXSETYY-YXKY--20240197). The guardians of the children all agreed and signed the informed consent form.

Sample Collection 

Samples were collected from three cases in each of the three groups, each with the same male and female composition and similar age, and 3-4 ml of fresh serum was stored in a -80°C freezer prior to use.

Exosome Extraction and Identification 

Exosome extraction: The exosomes were extracted by ultracentrifugation. For this process, the samples were quickly thawed at 37°C. The samples were then transferred to new centrifuge tubes and centrifuged at 2000 × g at 4°C for 30 min by a CP100MX ultracentrifuge (Hitachi, Ltd., Tokyo, Japan). Each supernatant was carefully transferred to a new centrifuge tube and centrifuged at 10,000 × g at 4°C for 45 min to remove large vesicles. The supernatants were filtered through 0.45 μm filter membranes, and the filtrates were collected. The filtrates were then transferred to new centrifuge tubes, a high-speed rotor was selected, and the samples were centrifuged at 100,000 × g at 4°C for 70 min. After the supernatants were removed, the pellets were resuspended in 10 mL of precooled 1× phosphate-buffered saline (PBS), and the samples were centrifuged again at 100,000 × g at 4°C for 70 min. Finally, the supernatants were removed, and the pellets were resuspended in 300 μL of precooled 1× PBS. A 20 μL aliquot was utilized for electron microscopy, a 10 µL aliquot was used for particle size determination, and the remaining exosomes were preserved at -80°C. 

Exosome identification: The morphology of the exosomes was observed by transmission electron microscopy (Hitachi); 10 μL of each sample was absorbed and deposited on a copper grid for one minute, and the liquid remaining after absorption was removed with filter paper. A 10 μL drop of uranyl acetate was added to the copper grid to precipitate for one minute, after which the remaining liquid was removed with filter paper, and the sample was dried at room temperature for several minutes. Then, an electron microscope was used for imaging at 100 kV. The particle size and concentration of the exosomes were detected by an N30E particle size analyzer (NanoFCM Inc., Xiamen, China), and the exosomes were diluted from 10 μL to 30 μL and tested with standard products after the instrument performance was qualified. Exosome-specific markers such as CD9, CD63, and CD81, and negative indicators such as Calnexin, were detected by Western blot. 

Synthesis of the First-Strand cDNA of miR-223 and miR-123 in Exosomes

Total RNA was extracted from exosomes with TRIzol reagent (Invitrogen 15596-026, USA), and the concentration and purity of the total RNA were detected by an ultraviolet spectrophotometer (SHIMADZU UV-2450, Japan) at wavelengths of 260 and 280 nm. The ratio of 260 nm/280 nm is between 1.8 and 2.0, which is an acceptable RNA purity. The total RNA was reverse-transcribed into cDNA using a first-strand cDNA synthesis kit (TaKaRa RR036B, Japan) (2 µg of total RNA in a 20 μL reaction mixture).

Detection of the Expression Levels of miR-223 and miR-123 in Exosomes by Reverse Transcription Polymerase Chain Reaction Primer Design

The sequences of miR-223, miR-132, and U6 were obtained from the National Center for Biotechnology Information (NCBI) databases. Primer Premier 5.0 (Premier Biosoft, CA, USA) was used to design primers. The upstream primer for miR-223 was 5'-ACACTCCAGCTGGGCGTGTATTTGACAAGC-3', and the downstream primer was 3'-TGGTGTCGTGGAGTCG-5'. The upstream primer for miR-132 was 5'-ACACTCCAGCTGGGACCGTGGCTTTCGATT-3', the downstream primer was 3'-TGGTGTCGTGGAGTCG-5'. The upstream primer for parameter U6 was 5'-CTCGCTTCGGCAGCACA-3', and the downstream primer was 3'-AACGCTTCACGAATTTGCGT-5'. 

Reverse transcription polymerase chain reaction (RT-PCR): The reaction system (total volume 20 μL) consisted of 10 μL of 2× real-time PCR master mix (SYBR Green), 1 μL of template (10´dilution of the cDNA), 2 μL of primer mix (F/R 10 μM), and 7 μL of 0.1% diethyl pyrocarbonate-treated water (DEPC). Products were detected by the stem ring method. The reaction procedure was as follows: predenaturation at 95.0°C for three min, denaturation at 95.0°C for five s, annealing at 55.0°C for 30 s, and extension at 72.0°C for 30 s. Forty-five cycles of amplification were performed, and a melting curve was generated after each amplification (95.0℃ for 15 s, 60.0℃ for one min, and 95.0℃ for 15 s). The reference genes U6 and miR-223, and miR-132 were amplified simultaneously in the three groups of samples. Appendix 1 shows that the melting curves of U6,miR-223, and miR-132 are all single peaks, indicating that their PCR products are all single specific amplifications, and no non-specific products or primer dimers are generated. Three replicates were performed for each sample of each gene, and the average values of the three tubes were calculated and analyzed. The relative expression levels of miR-223 and miR-132 were calculated using the formula 2^-ΔΔCt^, where ^ΔΔCt^=(^Ct^ target gene of sample ^- Ct^ reference gene of sample) Experimental group - (^Ct^ target gene of calibration sample ^-Ct ^reference gene of calibration sample) Control group.

Western Blot Analysis of STAT3 and SOCS3 Expression Levels in Exosomes

Protein extraction from exosomes: Lysate was added at a 1:1 ratio, placed on a shaking table platform at 4°C, shaken vigorously for 30 seconds, and placed on ice for four minutes; this process was repeated five times. The samples were centrifuged at 12,000 ´ g and 4°C for five minutes, after which the supernatant was collected. 

Protein quantification: The bicinchoninic acid (BCA) method was used for quantification. Eight protein amounts (0, 0.5, 1, 2, 4, 6, 8, and 10 μg) were selected to construct standard curves, and the working stock was prepared with 50:1 dilutions of stock A and stock B. Then, 0, 1, 2, 4, 8, 12, 16, and 20 μL of protein standard solution and 20 μL of diluted sample were added to an enzyme label plate, 200 μL of BCA working solution was added, and the mixture was thoroughly mixed and incubated at 37°C for 30 minutes. The standard curve no. 0 tube was used as a reference, and the solutions were evaluated at a wavelength of 562 nm. According to the absorption value of the measured sample, the corresponding protein content (μg) can be found on the standard curve, divided by the total volume of the sample diluent (20 μL), and multiplied by the dilution of the sample to determine the actual concentration of the sample (unit: μg/μL). 

Sodium dodecyl sulfate-polyacrylamide gel electrophoresis (SDS‒PAGE) electrophoresis and membrane transfer: The appropriate polyacrylamide gel concentration was selected according to the molecular weights of the target proteins; a 10% separation gel and a 5% concentration gel were prepared; the appropriate samples were added to the sample wells; a predyed protein marker was added to the well next to the samples; and 1´ SDS loading buffer was added to the wells without samples to maintain the balance of the gel surface. The power was turned on, the voltage was initially set to 60 V, and when the protein samples had entered the separation gel, the voltage was increased to 90 V. With reference to the position of the predyed marker, the electrophoresis was stopped when the target bands had entered the optimal separation zone of the gel (approximately 2/3 of the length of the gel). For Western blotting, the membrane transfer solution was precooled at 4°C in advance, the transfer tank of the apparatus was opened, and the inner surface was covered with a sponge cushion soaked with membrane transfer buffer solution. Three layers of Whatman 3MM (Cytiva, Buckinghamshire, United Kingdom) filter paper soaked with membrane transfer buffer solution were placed on the membrane cushion, and an NC membrane soaked with methanol and membrane transfer solution was placed on the filter paper. The glass plate encasing the gel was carefully pried open, the gel was placed in a tray containing membrane transfer solution, and the separation gel containing the target bands was removed. The samples were soaked in the transfer solution and placed on the NC membrane. Three layers of Whatman filter paper soaked in transfer buffer were placed on the separation gel, and the second sponge pad was placed on top so that the entire transfer sandwich consisted of the layers: fiber pad - filter paper - NC membrane - gel - filter paper - fiber pad. The transfer clip was closed, the transfer tank was placed in position, and the power supply of the Trans-Blot Turbo all-purpose protein transfer system was turned on. After transfer, the NC membrane was removed, marked, and washed with tris-buffered saline with Tween 20 (TBST) three times for five minutes each. 

Western blotting: Since CD63 was found to be stably expressed in exosomes in this study, CD63 was chosen as the loading control. The NC membrane was placed in a plate, a blocking solution containing 5% skim milk powder was added, and the plate was shaken for two hours. After blocking, the membrane was washed with TBST three times for five minutes each. The membrane was then placed in a plate containing primary antibody (Beringle) (rabbit anti-STAT3, rabbit anti-suppressors of cytokine signaling 3 (SOCS3), rabbit anti-CD9, rabbit anti-CD63, rabbit anti-CD81, and rabbit anti-Calnexin diluted with western primary antibody diluent, 1:1000) and incubated overnight on a shaking table at 4°C. On the second day, the membrane was removed, oscillated at room temperature for 30 min, after which the primary antibody was removed, and the membrane was washed with TBST three times for 10 min each. The secondary antibody was diluted with diluent (sheep anti-rabbit IgG-HRP, 1:2000) and added to the membrane, which was then oscillated in a room temperature shaker for two hours. After the secondary antibody reaction, the secondary antibody was removed. Then, the membrane was washed with TBST three times for five minutes each. The A and B liquids in an enhanced chemiluminescence (ECL) kit were mixed at a volume of 1:1 and used as the working stock. The NC membrane was removed from the TBST, the excess liquid was shaken off, the membrane was placed face up on plastic wrap, an appropriate amount of ECL working stock was added, and the membrane was covered with plastic wrap. A ChemiDoc MP Imaging System (Bio-Rad ChemiDoc MP Imaging System, Bio-Rad Laboratories, Inc., CA, USA) was used for imaging, and Gel-Pro32 (Media Cybernetics, L.P., Maryland, USA) software was used to analyze the results.

Statistical processing and analysis of data

IBM SPSS Statistics for Windows, Version 21 (Released 2012; IBM Corp., Armonk, New York, United States). was used for the statistical analysis. Normally distributed data, such as expression levels of miR-223 and miR-132 in exosomes, were expressed as the mean ± standard deviation, and one-way ANOVA was used for comparisons among groups. A skewed distribution of data, such as expression levels of IL-6, IL-8, IL-10, and IL-17 in serum, was tested by the rank sum test, represented by the median (P25, P75), and the independent sample Kruskal‒Wallis test was used for comparisons among multiple groups. Spearman correlation analysis was used to analyze correlations between variables. P<0.05 was considered to indicate statistical significance.

## Results

General information 

In the Rheumatology and Immunology Department of Jiangxi Children's Hospital, 60 patients with sJIA (sJIA group), including 30 males and 30 females, aged 8.65±3.90 years, were selected from September 2022 to December 2023. Around 23 patients with aJIA (aJIA group), including 13 males and 10 females, aged 9.10±3.40 years, were also selected. In addition, 20 healthy individuals (healthy control group, NC group), 10 males and 10 females, aged 8.47±2.65 years, were selected from the child health care department of our hospital during the same period. There were no significant differences in sex or age among the three groups (P>0.05). The results are shown in Table 1.

**Table 1:** General information Normally distributed data are expressed as the mean ± standard deviation, and one-way ANOVA was used for comparisons among groups. sJIA: systemic juvenile idiopathic arthritis; aJIA: articular juvenile idiopathic arthritis; NC: normal control

Index	sJIA group (n=60)	aJIA group (n=23）	NC group (n=20）	P-value
Female (%)	30 (50%)	10 (43.48%)	10 (50%)	/
Age (year)	8.79±2.82	10.37±3.10	9.10±3.96	0.351

Identification of exosomes 

**Figure 1:**
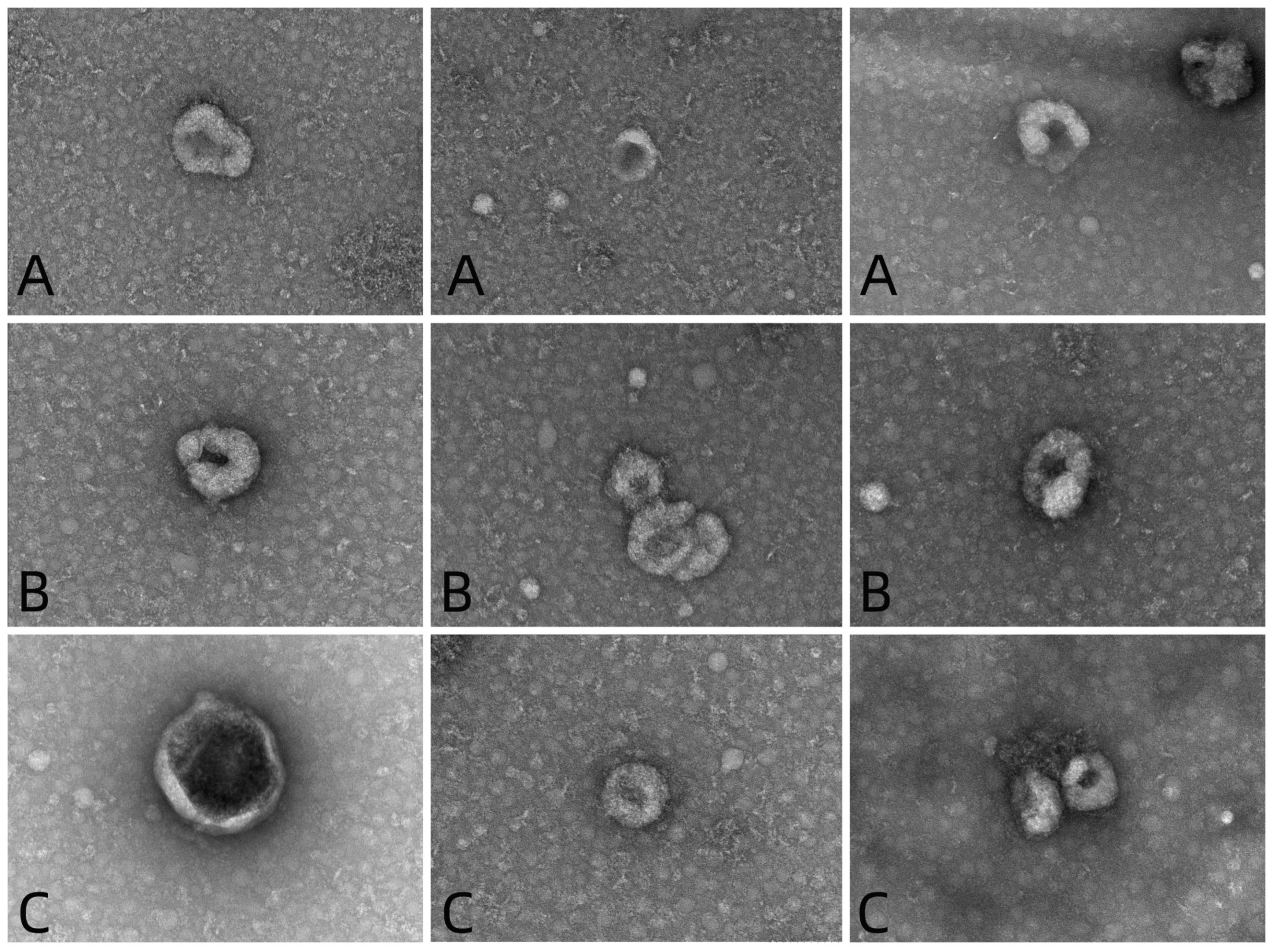
Electron microscopy images of exosomes Exosomes were observed under electron microscopy. The extracted exosomes were membranous vesicles with diameters of approximately 30-150 nm. The magnification of all pictures was x40.0k, and the horizontal length of all pictures corresponded to the actual size of 100nm. A: electron microscopy images of exosomes in the sJIA group; B: electron microscopy images of exosomes in the aJIA group; C: electron microscopy images of exosomes in the NC group. sJIA: systemic juvenile idiopathic arthritis; aJIA: articular juvenile idiopathic arthritis; NC: normal control

**Figure 2:**
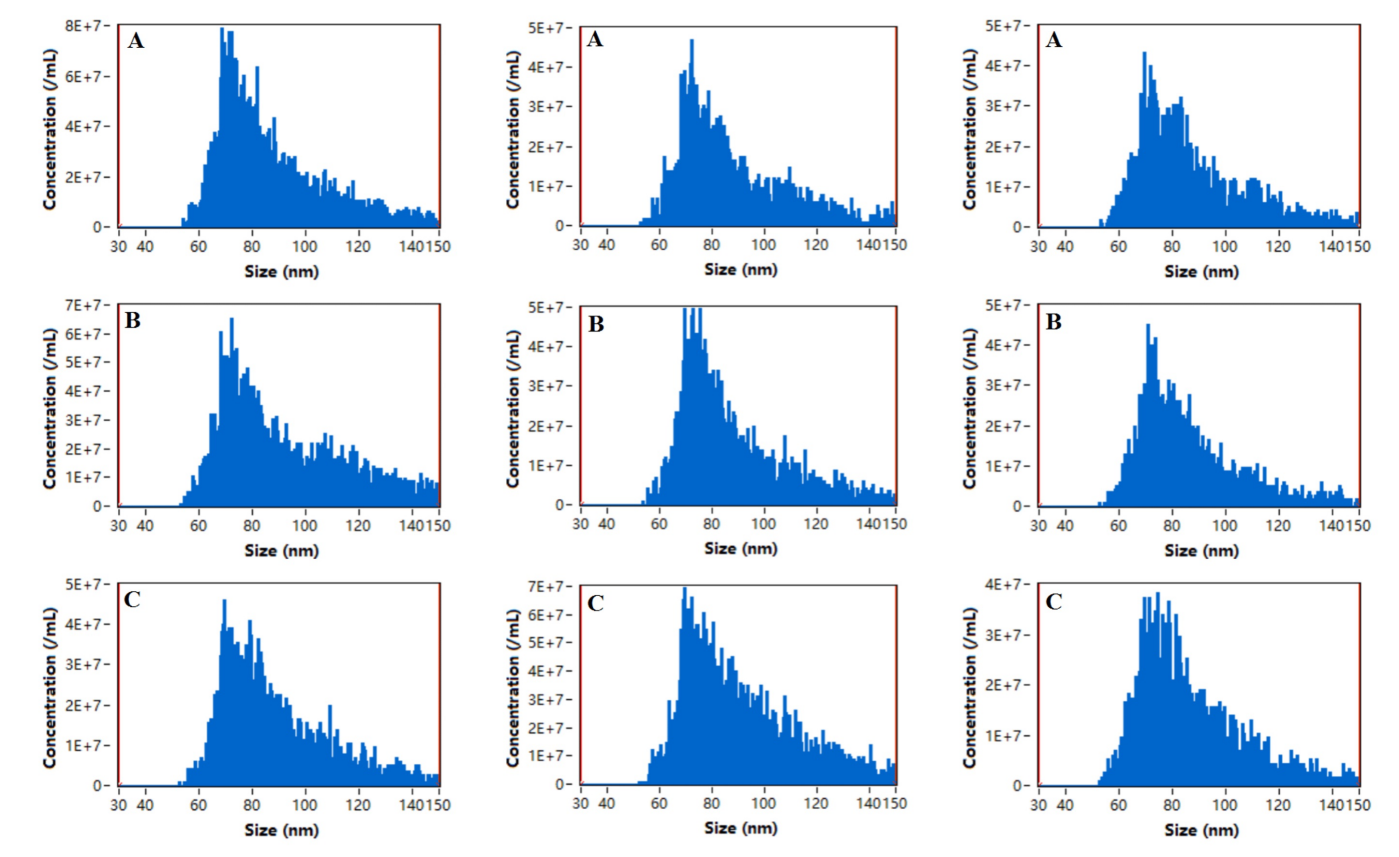
Particle size distributions and concentrations of exosomes The particle size and concentration of the exosomes were detected by an N30E particle size analyzer, and the exosomes were diluted from 10 μL to 30 μL and tested with standard products after the instrument performance was qualified. The horizontal coordinate of all the pictures in the figure represents the particle size of exosomes, and the vertical coordinate represents the concentration of exosomes. A: in the sJIA group, particle size distributions are respectively 87.3 nm, 87.7 nm, and 86.7 nm; concentrations of exosomes are respectively 4.07 × 10⁹ particles/mL, 2.17 × 10⁹ particles/mL, and 2.16 × 10⁹ particles/mL; B: in the aJIA group, particle size distributions are respectively 91.9 nm, 87.7 nm, and 86.5 nm; concentrations of exosomes are respectively 3.88 × 10⁹ particles/mL, 2.63 × 10⁹ particles/mL, and 2.09 × 10⁹ particles/mL; C: in the NC group, particle size distributions are respectively 88.6 nm, 90.4 nm, and 86.7 nm; concentrations of exosomes are respectively 2.63 × 10⁹ particles/mL, 4.48 × 10⁹ particles/mL, and 2.25 × 10⁹ particles/mL. sJIA: systemic juvenile idiopathic arthritis; aJIA: articular juvenile idiopathic arthritis; NC: normal control

**Figure 3:**
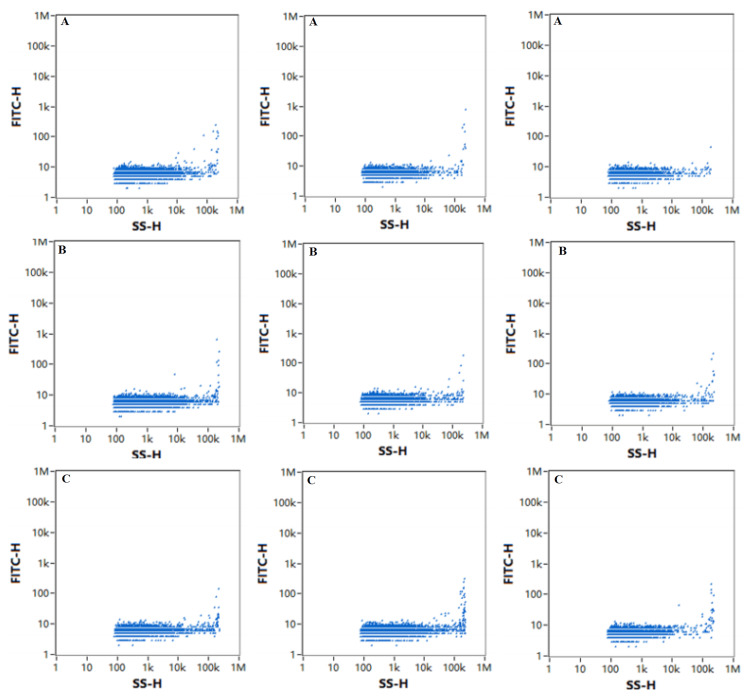
Particle position distribution of exosomes Particle position distributions were presented during particle size distributions and concentration of exosome detection by the N30E particle size analyzer. The horizontal coordinate represents lateral scattered light. All particles are in the negative fluorescence group because they are not fluorescently stained. A: particle position distribution of exosomes in the sJIA group; B: particle position distribution of exosomes in the aJIA group; C: particle position distribution of exosomes in the NC group. sJIA: systemic juvenile idiopathic arthritis; aJIA: articular juvenile idiopathic arthritis; NC: normal control

**Figure 4:**
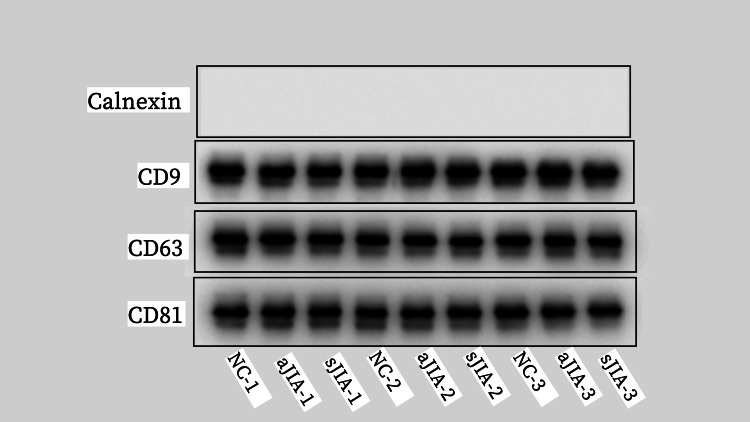
Specific markers of exosomes Exosome-specific markers such as CD9, CD63, and CD81, and negative indicators such as calnexin, were detected by Western blot. The first sample in the sJIA group (sJIA-1); the second sample in the sJIA group (sJIA-2); the third sample in the sJIA group (sJIA-3); the first sample in the aJIA group (aJIA-1); the second sample in the aJIA group (aJIA-2); the third sample in the aJIA group (aJIA-3); the first sample in the NC group (NC-1); the second sample in the NC group (NC-2); and the third sample in the NC group (NC-3). sJIA: systemic juvenile idiopathic arthritis; aJIA: articular juvenile idiopathic arthritis; NC: normal control

The extracted exosomes were membranous vesicles with diameters of approximately 30-150 nm, and the concentrations were within the normal range. The extracted exosomes expressed CD9, CD63, and CD81, while Calnexin was not expressed. The exosomes were successfully extracted. The results are shown in Figures 1-4.

Expression levels of miR-223 in exosomes

**Figure 5:**
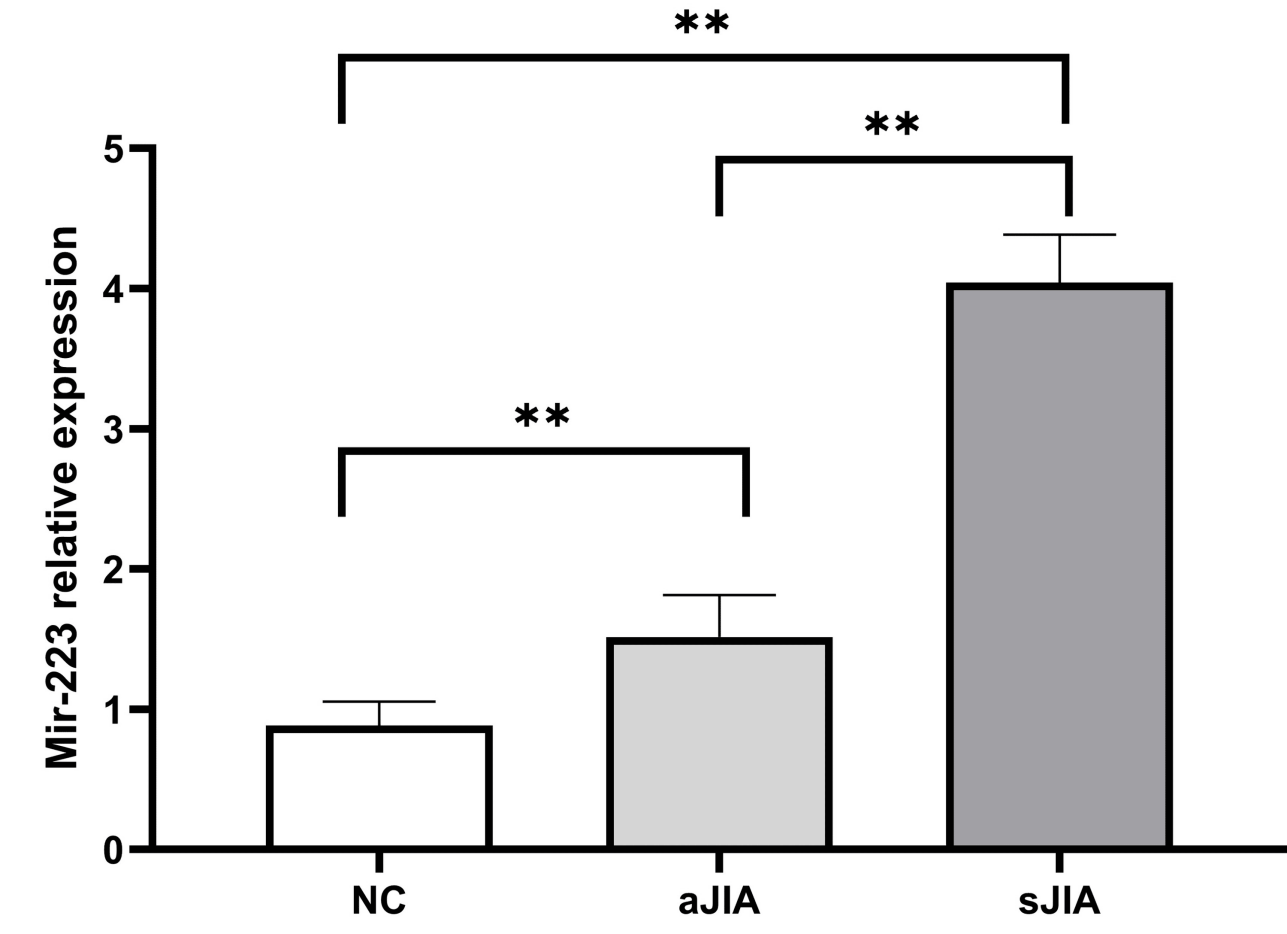
Expression levels of miR-223 in exosomes sJIA: systemic juvenile idiopathic arthritis; aJIA: articular juvenile idiopathic arthritis; NC: normal control **P<0.001

The expression level of miR-223 in serum exosomes in the sJIA group was significantly greater than that in the aJIA and NC groups (4.04±0.34 vs. 1.52±0.30, 0.88±0.17), and the differences were statistically significant (P<0.001). In addition, the expression level of miR-223 in serum exosomes in the aJIA group was significantly greater than that in the NC group (P<0.001) (Figure 5).

Expression levels of miR-132 in exosomes 

**Figure 6:**
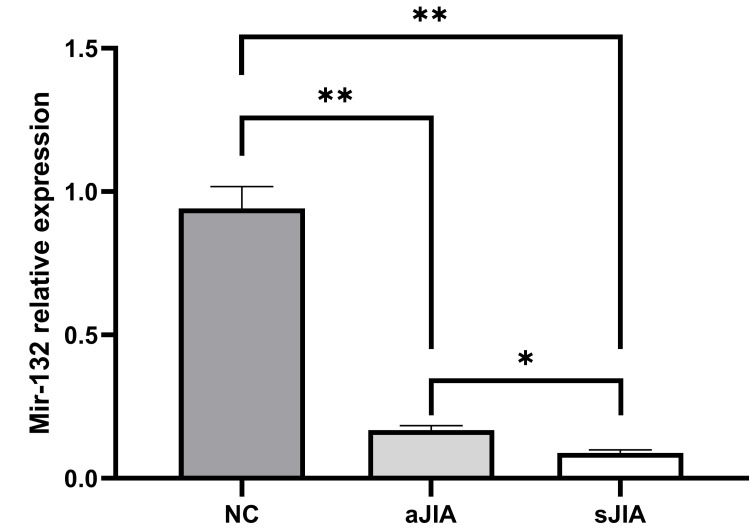
Expression levels of miR-132 in exosomes sJIA: systemic juvenile idiopathic arthritis; aJIA: articular juvenile idiopathic arthritis; NC: normal control **P<0.001, *P<0.05

The expression level of miR-132 in serum exosomes in the sJIA group was significantly lower than that in the aJIA and NC groups (0.09±0.01 vs. 0.17±0.02, 0.94±0.08), and the differences were significant (P<0.001). In addition, the expression level of miR-132 in serum exosomes in the aJIA group was significantly lower than that in the NC group (P<0.001) (Figure 6).

Expression levels of IL-6, IL-8, IL-10, and IL-17 in serum from the sJIA, aJIA, and NC groups 

The expression levels of IL-6, IL-8, and IL-10 in the serum from the sJIA group were significantly greater than those in the serum samples from the aJIA and NC groups (P<0.001). The expression levels of IL-6, IL-8, and IL-10 in the serum from the aJIA group were significantly greater than those in the serum from the NC group (P<0.001). The IL-17 expression in the sJIA group was significantly greater than the expression levels in the aJIA and NC groups (P<0.001). There was no significant difference in the serum levels of IL-17 between the sJIA and aJIA groups (P>0.05) (Table 2).

**Table 2:** Expression levels of IL-6, IL-8, IL-10, and IL-17 The measurement data conforming to a skewed distribution were tested by the rank sum test, represented by the median (P25, P75). P value labels: ★P<0.001 vs. the NC group; ●P<0.001 vs. the aJIA group. sJIA: systemic juvenile idiopathic arthritis; aJIA: articular juvenile idiopathic arthritis; NC: normal control

Index	sJIA group M (P25,P75)	aJIA group M (P25,P75)	NC group M (P25,P75)	H value	P-value
IL-6	317.62(214.74,480.50)^★^^●^	80.60(69.23,122.87)^★^	2.54(2.25,2.71)	69.78	0.000
IL-8	1006.50(528.75,1802.25)^★^^●^	448.00(226.00,721.00)^★^	8.38(6.69,10.19)	56.55	0.000
IL-10	34.69(26.92,67.33)^★^^●^	10.32(8.13,16.23)^★^	1.58(1.40,1.69)	47.61	0.000
IL-17	14.00(10.88,21.88)^★^	12.05(6.42,19.29)^★^	2.27(2.17,2.42)	49.99	0.000

Correlations between the expression levels of miR-223 and miR-132 in exosomes and the clinical inflammatory indicators IL-6, IL-8, IL-10, and IL-17 

**Figure 7:**
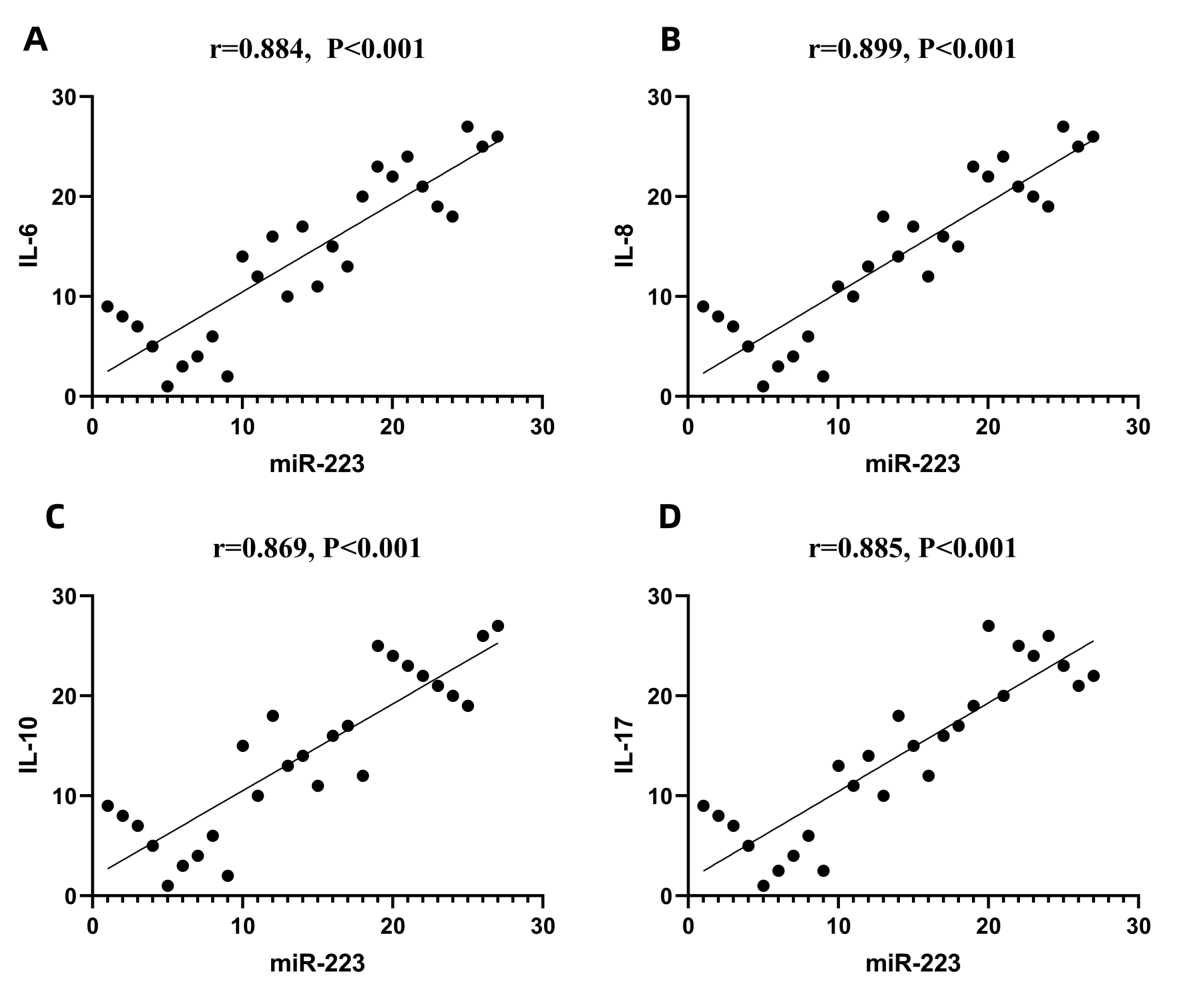
Correlations between miR-223 and IL-6, IL-8, IL-10, and IL-17 Spearman correlation analysis was used to analyze correlations between variables. The values of miR-223 and interleukin were sorted respectively and assigned ranks, with each point representing a rank pair of an observed value. A: the expression level of miR-223 in exosomes was positively correlated with the expression levels of IL-6, r value was 0.884, P<0.001; B: the expression level of miR-223 in exosomes was positively correlated with the expression levels of IL-8, r value was 0.899, P<0.001; C: the expression level of miR-223 in exosomes was positively correlated with the expression levels of IL-10, r value was 0.869, P<0.001; D: the expression level of miR-223 in exosomes was positively correlated with the expression levels of IL-17, r value was 0.885, P<0.001. miRNA-223: miR-223

**Figure 8:**
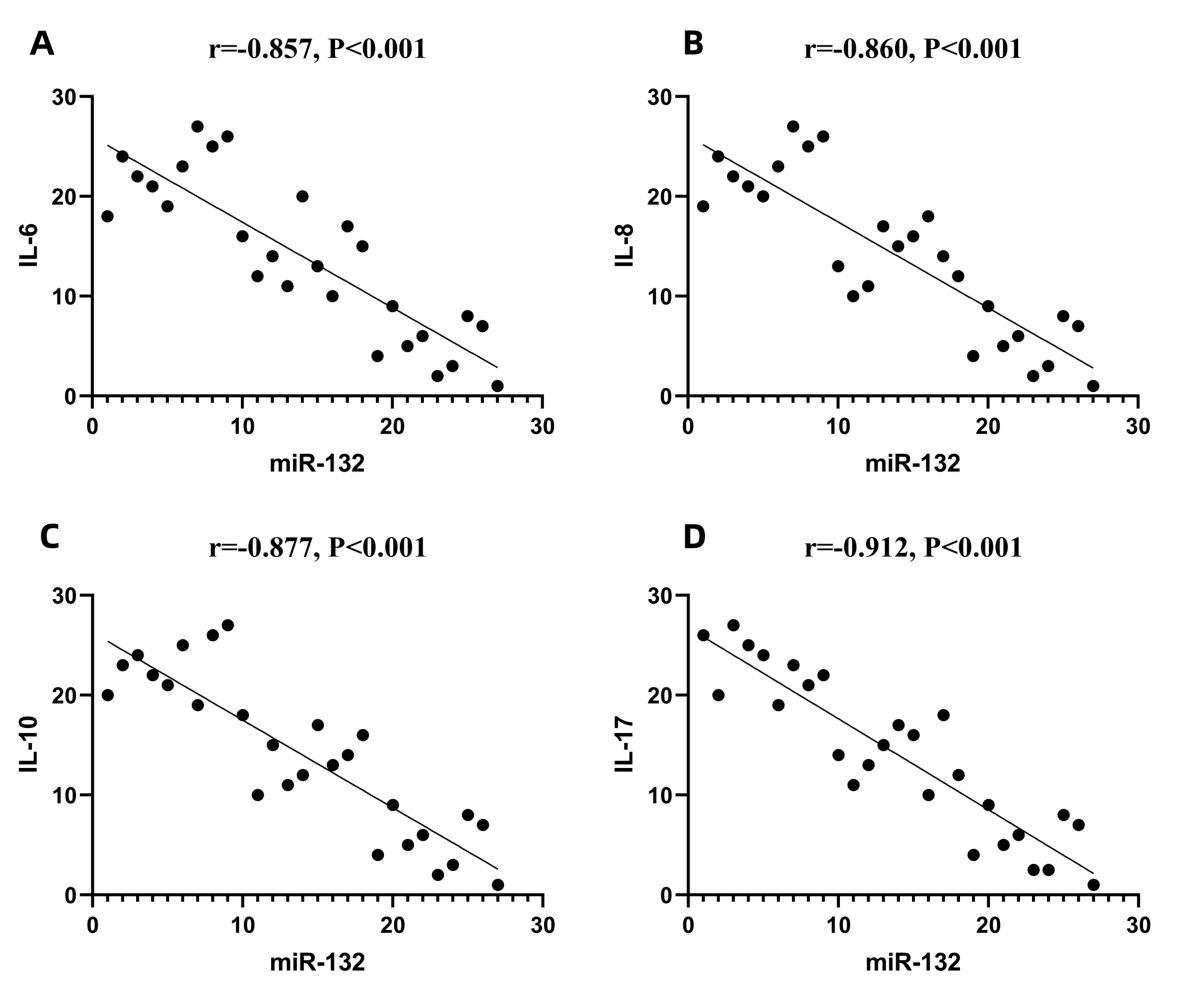
Correlations between miR-132 and IL-6, IL-8, IL-10, and IL-17 Spearman correlation analysis was used to analyze correlations between variables. The values of miR-132 and interleukin were sorted respectively and assigned ranks, with each point representing a rank pair of an observed value. A: the expression level of miR-132 in exosomes was negatively correlated with the expression levels of IL-6; the r value was -0.857, P<0.001; B: the expression level of miR-132 in exosomes was negatively correlated with the expression levels of IL-8, r value was -0.860, P<0.001; C: the expression level of miR-132 in exosomes was negatively correlated with the expression levels of IL-10, r value was -0.877, P<0.001; D: the expression level of miR-132 in exosomes was negatively correlated with the expression levels of IL-17, r value was -0.912, P<0.001. miRNA-132: miR-132

The expression level of miR-223 in exosomes was positively correlated with the expression levels of the clinical inflammatory indicators IL-6, IL-8, IL-10, and IL-17 (P<0.001). The expression level of miR-132 in exosomes was negatively correlated with the expression levels of the clinical inflammatory indicators IL-6, IL-8, IL-10, and IL-17 (P<0.001) (Figures 7, 8).

SOCS3 protein relative expression levels 

**Figure 9:**
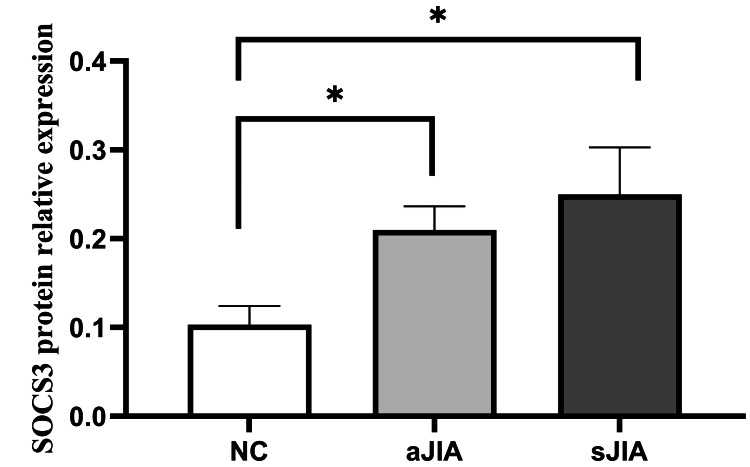
SOCS3 protein relative expression levels The relative expression of SOCS3 protein is represented by the ratio of gray values, which is the ratio of the gray value of SOCS3 to the gray value of CD63. sJIA: systemic juvenile idiopathic arthritis; aJIA: articular juvenile idiopathic arthritis; NC: normal control *P<0.05.

**Figure 10:**
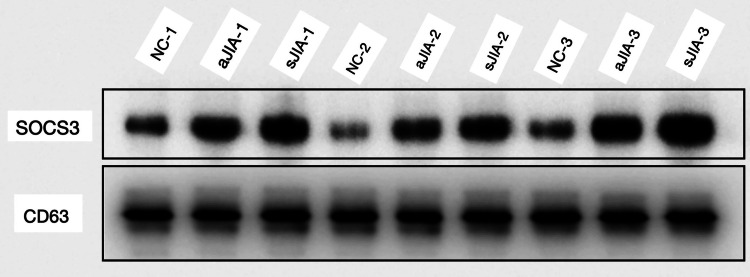
Western blot image of SOCS3 Since CD63 was found to be stably expressed in exosomes in this study, CD63 was chosen as the loading control. The first sample in the sJIA group(sJIA-1); the second sample in the sJIA group(sJIA-2); the third sample in the sJIA group(sJIA-3); the first sample in the aJIA group(aJIA-1); the second sample in the aJIA group(aJIA-2); the third sample in the aJIA group(aJIA-3); the first sample in the NC group(NC-1); the second sample in the NC group(NC-2); the third sample in the NC group(NC-3). sJIA: systemic juvenile idiopathic arthritis; aJIA: articular juvenile idiopathic arthritis; NC: normal control

The relative expression levels of SOCS3 in both the sJIA and aJIA groups were significantly greater than those in the NC group (0.25±0.05 and 0.21±0.03 vs. 0.10±0.02, respectively), and the differences were statistically significant (P<0.05). However, there was no significant difference in the SOCS3 relative expression levels between the sJIA and aJIA groups (P>0.05) (Figures 9, 10). There were no significant differences in STAT3 relative expression in exosomes among the three groups (P>0.05).

## Discussion

miRNAs isolated from patients' biological samples, including serum, urine, and exosomes of serum and urine, are novel biomarkers and are involved in the pathogenesis of many human diseases, including cancer, metabolic diseases, neurological disorders, and autoimmune diseases [19,20]; these miRNAs have become a current research hotspot. miR-223 is upregulated in many diseases. For example, Lorenzo Agostino Citterio et al. [21] reported that the expression levels of miR-7-1-5p and miR-223-3p in the serum and exosomes of patients with Parkinson's disease were upregulated and found that they were involved in the occurrence of neuroinflammation and played important roles in patients with Parkinson's disease. Liyan Dou et al. reported that the overexpression of miR-223-5p in non-small cell lung cancer could significantly reduce the mRNA and protein expression levels of the key transcription factor E2F8 [22]. However, the expression of miR-132 tends to decrease in some diseases. For example, Hannah Walgrave et al. [23] reported that miR-132 was expressed at low levels in patients with Alzheimer's disease. If the expression of miR-132 was restored, hippocampal nerve remodeling and memory loss could be restored. Milad Rafat et al. reported that miR-132 expression was significantly reduced in various malignant tumors, such as liver, breast, and stomach cancers [24]. Studies [25,26] have also reported that miRNAs play important roles in the regulation of the immune response in JIA, with particular attention given to them as potential biomarkers for diagnosis/prognosis. Xinyi Wei et al. [27] reported that human umbilical cord mesenchymal stem cells (HUCMSCs)-derived exosomes (HUCMSCs-Exos) delivered miR-29-3p targeting AhR expression to inhibit IL-22 in JlA-CD4+ T cells through alleviating arthritic synovial fibroblast activation. Studies [7] have reported that the expression levels of miR-16, miR-146a, and miR-223 in the serum of patients with JIA were significantly greater than those in healthy controls, while miR-132 expression was decreased. To our knowledge, in this study, for the first time, the expression level of miR-223 in the serum exosomes of sJIA patients was noted to be significantly greater than that in the serum exosomes of the aJIA and NC groups. The expression level of miR-223 in serum exosomes in the aJIA group was also significantly greater than that in the NC group. However, the expression level of miR-132 in the serum exosomes of the sJIA group was significantly lower than that in the serum exosomes of the aJIA and NC groups. The expression level of miR-132 in serum exosomes in the aJIA group was also significantly lower than that in the NC group, which was consistent with research results reported by others, both domestically and internationally. Therefore, miR-223 and miR-132 in exosomes may serve as two potential novel markers of JIA.

As mentioned previously, continuous overactivation of the JAK/STAT signaling pathway causes JIA patients to produce large numbers of proinflammatory factors, such as IL-1, IL-6, IL-8, and IL-10, ultimately leading to a hyperinflammatory response. This response is one of the important factors in the pathogenesis of JIA [4,5]. Therefore, we measured the differences in the expression levels of various inflammatory factors between JIA patients and healthy controls and analyzed the correlations between the expression of miR-223, miR-132, and various inflammatory factors. We found that the serum expression levels of IL-6, IL-8, and IL-10 in the sJIA group were significantly greater than those in the aJIA and NC groups. The serum levels of IL-6, IL-8, and IL-10 in the aJIA group were also significantly greater than those in the NC group. The IL-17 expression in the serum of the sJIA and the aJIA groups was significantly greater than the expression levels in the NC groups, but there was no significant difference in IL-17 expression in the serum of the sJIA group compared with that in the aJIA group. Moreover, the expression level of miR-223 in exosomes was positively correlated with the clinical inflammatory indicators IL-6, IL-8, IL-10, and IL-17. However, the expression level of miR-132 in exosomes was negatively correlated with the clinical inflammatory indicators IL-6, IL-8, IL-10, and IL-17. Therefore, it is speculated that miR-223 and miR-132 may play very important roles in the inflammatory response in JIA.

Zeinab Amini-Farsani et al. [28] reported that several miRNAs, especially miR-9, miR-98, miR-223, and miR-214, play key roles in the regulation of the NF-kB and JAK-STAT signaling pathways as inflammatory regulators. The JAK/STAT pathway activates the transcription of many genes in the inflammatory pathway, including ACE2, IL-8, IL-6, IL-2, IL-17, IL-1β, CSF3, CSF2, CCL3, TNF-α, and IFN-γ, which are regulated by different miRNAs [29]. It has also been shown [30] that miR-223 regulates the expression of the inflammatory cytokines IL-6 and STAT3. STAT3 is the core mediator in the pro-inflammatory cytokine signaling pathway and is involved in the pathological changes of synovial tissue. In patients with JIA, the activity or expression level of STAT3 shows an abnormally elevated state [5]. However, this study revealed no significant difference in STAT3 expression levels between JIA patients and healthy controls, which was inconsistent with the findings of some previous reports. The reasons are as follows: First, the STAT3 detected in this study originated from serum exosomes, but STAT3 is mainly located within cells, with extremely low content in serum. It was mostly detected in synovial fluid or mononuclear cells in most studies. Second, it is the activated form of STAT3, namely phosphorylated STAT3 (P-STAT3) rather than STAT3 in JIA. In the future study, we will detect both P-STAT3 and STAT3 simultaneously. The targets of the miRNAs are the highly conserved 3'UTRs of several signaling molecules related to JAK/STAT, including SOCS, such as SOCS1, SOCS3, and SOCS5 [8]. SOCS proteins are feedback suppressors in the JAK/STAT signaling pathway, and through various mechanisms, SOCS proteins negatively regulate the innate and adaptive immune responses [31]. SOCS3 leads to the inhibition of STAT3 phosphorylation by binding to JAK and cytokine receptors, resulting in a protective immune response against infectious and inflammatory diseases [32]. Wenbo Zhang et al. [33] reported that SOCS3 protein levels in JIA patients were significantly increased, which was consistent with the SOCS3 protein expression levels observed in this study. Thus, miR-223 might promote a hyperinflammatory response in JIA patients and might enhance the activation of the JAK/STAT signaling pathway. While miR-132 may inhibit the high inflammatory response in JIA, its inhibitory effect may be closely related to SOCS3 through the JAK/STAT signaling pathway.

Limitations of the study

Our study had several limitations. Our sample size was relatively small, which may limit the generalizability of the findings. Second, the working curves of miR-223 and miR-132 in JIA could not be evaluated due to the small number of cases with these conditions. Lastly, more detailed and specific mechanisms of miR-223 and miR-132 in the persistent overexpression of inflammation in JIA were not studied in this paper. 

## Conclusions

To sum up, in this study, the increased expression of miR-223 in the exosomes of JIA patients and the decreased expression of miR-132 were closely related to the persistent inflammatory response in JIA patients. We speculated that miR-223 and miR-132 may be two potential new markers of JIA, providing new ideas for diagnostic tests and therapeutic interventions. But larger studies are needed to confirm these findings and assess their generalizability across diverse populations. miR-223 may promote inflammation in JIA patients by enhancing the JAK/STAT signaling pathway, while miR-132 may reduce inflammation in JIA patients by inhibiting the JAK/STAT signaling pathway. Its inhibitory effect may be closely related to SOCS3 through the JAK/STAT signaling pathway. However, it is not yet understood how miR-223 and miR-132 interact with STAT3 and SOCS3 in the exosomes of JIA patients. Further research is needed to obtain more definitive evidence of their interactions.

## Figures and Tables

**Figure 11:**
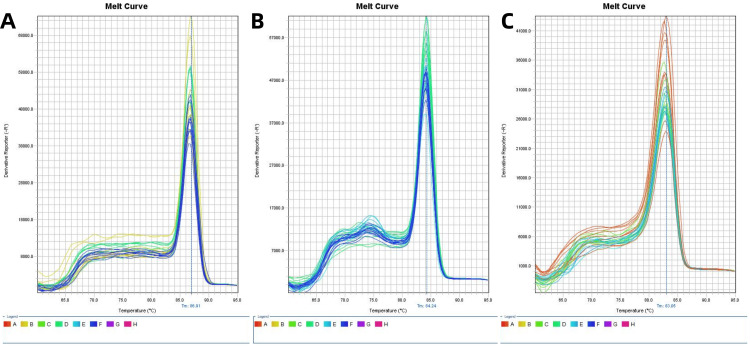
Melt curve of PCR A: the melt curve of miR-223; B: the melt curve of miR-132; C: the melt curve of U6 PCR: polymerase chain reaction

## Appendices

Appendix 1 
